# The Poor Babies

**Published:** 1876-08

**Authors:** 


*TWENTY-THIRD YEAR OF*

### HALL'S JOURNAL OF HEALTH.

***E. H. Gibbs, M.D., Editor.***

VOL. XXIII.] AUGUST, 1876. [NO. 8.

### *THE POOR BABIES.*

Two thousand little children died in the City of New York during the first twenty-five days of July, 1876, of what are *erroneously* called "Bowel Complaints." What an army of little martyrs to the *noble science* of medicine! In view of such facts and such figures, repeated year after year, with the regularity of the seasons, without hope or promise of amelioration, we need not wonder that a majority of the best people in this country have lost confidence in the regular medical profession, and in confusion are drifting off toward quackery, preferring to accept that with all its ignorant absurdities to the tortures of what is understood as the scientific practice of medicine. No one can accuse us of habitual fault-finding. But the writhing forms of a hundred dying infants each day appeal to every human being to aid in checking this wicked "slaughter of the innocents."

It was with a shudder that we read, a few days ago, that the "Board of Health" had appointed fifty additional physicians—with *carte blanche* to order drugs—to attend the poor children. Fifty more wolves turned into the sheep-fold. Alas! poor babies. Who, without being told, would not have known from the result that the number of your destroyers had increased?

Had the "Board of Health" possessed the good sense to appoint fifty women of experience to nurse these little sufferers, the death roll would have been shorter. We hope the "Board of Health" was not appointed as some "Boards" seem to be, with especial reference to its incompetency. "By their works shall ye know them." We do not wish to be understood as censuring all physicians for their method of treating "cholera infantum." They all have the highest medical authority for using the most potent and dangerous drugs in these cases, but there are very many who have brains and make good use of them. They don't allow *popular* medical authors to do *all* their thinking. They are guided by reason, and they profit by experience.

Of the two thousand children who died of "cholera infantum" in the City of New York during July, very many, no doubt, inherited constitutions so depraved that no care would have saved them. Others died from the depressing influence of filthy surroundings. These should have been removed to healthier localities, and *properly* treated and nursed ; but by far the largest proportion of the two thousand victims, we verily believe, were poisoned by the various preparations of OPIUM. The stubbornness with which a majority of respectable physicians adhere to this murderous practice is astonishing. The fatal error arises from their supposing "cholera infantum" to be a disease of the bowels. Nearly all *American* medical writers assert that it is, and they don't take the trouble to investigate for themselves. Now, "cholera infantum" is no more a disease of the bowels than it is of the feet. In fact, it is not a disease at all until it is made so by injudicious medical treatment and improper care. It is simply a TORPID CONDITION of the LIVER, following any irritating cause, as teething, sultry weather, foul air, indigestible food, and insufficient clothing. If the irritating causes are removed—which *may* be done by removing the child to healthier localities—recovery will almost always follow, even without the use of medicine. It is safer, however, to treat the child, directing the treatment to the liver. So soon as the liver acts, the bowels will assume their natural condition, and complete recovery will speedily follow. "Soothing Syrups" or other preparations of opium, will inevitably make trouble, by checking or arresting the discharges from the bowels, and thus causing congestion of the brain or lungs, from which nearly all these fatal cases arise. All assertions to the contrary, there is as large a per centage of cases of "cholera infantum" to population in the country as in the city, and they are just as fatal, owing to the OPIUM treatment. The statement is frequently made, that "cholera infantum" is hardly known in Europe. We happen to know that this is a mistake, arising, probably, from the fact that such cases are judiciously treated and are seldom fatal. Hence, we do not hear of them.

There will be a larger proportion of deaths among children than adults in crowded and filthy portions of great cities, until the sanitary condition of such localities is greatly improved. But that children of sound constitutions, with home comforts and good nursing, should ever die of "cholera infantum" is a reproach and a disgrace to the medical profession. Without their ignorant interference, deaths from this cause would be rare, if good nursing and good food were substituted.

It is a mistake to suppose that only the children of the poor and the miserable die from the causes above stated. The proportion of deaths among children in the higher ranks of life is fearful. The parents of such children are very often ignorant of what is essential to the health and well being of their offspring. Their little ones are placed in charge of ignorant, often brutal nurses, with instructions to give them barely food enough to sustain life, and to dress them more with reference to fashion than to health and comfort. When the child sickens, it is not restored to health by the affectionate care of the mother, as in former times, but the family physician is called ; and if it dies, it is by the *dispensation of Providence.* The children of poverty and of misery are not the only victims of malpractice.

